# Critical Issues in the Management of Newborn Hearing Screening in the Time of COVID-19 in Umbria, Italy

**DOI:** 10.3390/children9111736

**Published:** 2022-11-11

**Authors:** Valeria Gambacorta, Eva Orzan, Egisto Molini, Ruggero Lapenna, Matteo Paniconi, Alfredo Di Giovanni, Mario Faralli, Giampietro Ricci

**Affiliations:** 1 Section of Otorhinolaryngology, University of Perugia, 06129 Perugia, Italy; 2 Audiology and Otorhinolaryngology Unit, Institute for Maternal and Child Health-IRCCS “Burlo Garofolo”, 34137 Trieste, Italy

**Keywords:** universal newborn hearing screening, benchmarks, hearing loss

## Abstract

Hearing impairment is the most frequent of the sensorial defects in humans, and if not treated promptly, can severely impair cognitive and spoken language skills. For this reason, a universal newborn hearing screening (UNHS) has been established. The purpose of our study is to examine, by means of a retrospective analysis, the results of the UNHS program in the Umbria region during the spread of COVID-19 (2020–2021), comparing the same data from the years 2011–2012, to understand if the program has improved. Our study has shown how the coverage rate of well born babies’ (WB) screening has significantly increased to currently meet the JCIH benchmark. The percentage of WB referrals significantly decreased in 2020–2021, another indicator of the screening program’s greater efficiency in Umbria. However, a critical issue has emerged: the percentage of those lost to follow-up is greater than 30%, well above the benchmark. As far as the COVID-19 pandemic has certainly had a significant impact, it is necessary to carefully monitor those who do not access the diagnostic level. To emphasize the importance of a proper screening program, it will be helpful to strengthen the computerized data collection system and create an information network between audiologists, pediatricians and families.

## 1. Introduction

Hearing impairment is the most frequent of the sensorial defects in humans. The incidence of sensorineural hearing loss ranges from 1 to 2 per 1,000 live births [1,2]. Severe or profound deafness present at birth, if not diagnosed and treated promptly, can severely impair cognitive and spoken language skills. Communication, relational and affective skills are affected, preventing easy integration into school and society [3,4,5,6]. For this reason, a universal newborn hearing screening (UNHS) has been established, to ensure an early diagnosis [7,8]. The Joint Committee on Infant Hearing (JCIH) in a 2019 position statement established an early hearing detection and intervention (EHDI) program in order to identify infants with permanent hearing loss as early as possible. This position statement recommended UNHS by 1 month of age, identification of hearing loss by 3 months and early intervention by 6 months of age, setting the following benchmarks: (1) screening coverage of 95% or more; (2) missed/lost less than 10%; (3) refer (patients who fail the screening test) less than 4% [9].

Umbria Region, with the Regional Council Resolution no. 789 of 21 May 2007, has approved the UNHS consisting of two strategies: (1) otoacoustic emissions (TEOAE) 24 h after birth for newborns without audiological risk factors (well born babies—WB). WB who fail the first test (REFER) are sent to a second-level center to undergo a second-level screening with an automatic auditory evoked potential of the brainstem (AABR) test within three months of birth. WB REFER at the second level are subsequently sent to a third-level center to undergo a diagnostic examination with the administration of an auditory brainstem evoked potential (ABR) test. (2) TEOAE + AABR 24 h after birth for babies with identified audiological risk factors (BRF). BRF REFER at TEOAE and AABR, or BRF PASS at TEOAE and REFER at AABR are sent to a third-level center to undergo a diagnostic examination with ABR. This screening program has been recently improved (October 2019) by developing an electronic database, which is shared among all seven regional birth centers. Thanks to this method, we can monitor all newborns’ hearing screening data. Patients who fail the initial screening levels or those with risk factors are managed at the regional third-level center according to the guidelines. The regional database allows low referral rates, high follow-up rates, high coverage and early intervention. 

In our study, we examined the UNHS in the Umbria region during the spread of coronavirus disease 2019 (COVID-19), which had a strong impact on the national health system, resulting in negative consequences for patient outcomes in the future. Even if UNHS was one of the non-postponable procedures, in some cases its correct execution was nevertheless affected by the state of a public health emergency.

The purpose of this study is to examine the results of the UNHS program in the Umbria region in the two-year period 2020–2021, comparing the same data from the years 2011–2012 [10], to understand which aspects of the program have improved and which still need to be improved.

## 2. Materials and Methods

We performed a retrospective analysis of UNHS performed in the Umbria region between January 2020 and December 2021. We have included in the study both BRF and WB babies. Babies with audiological risk factors were identified following the guidelines proposed by the JCIH in the 2019 position statement [9]. As indicated by the guidelines, all babies have been screened 24 h after birth, the WB have been subjected to otoacoustic emissions (TEOAE) tests, while BRF were subjected to TEOAE + automatic auditory evoked potential of the brainstem (A-ABR) screening. The A-ABR screening was performed for WB who failed the initial TEOAE test. WB and BRF who both failed screening tests (TEOAE and A-ABR) were then addressed to the regional referral center for pediatric audiology. The data are compared with equivalent data from 2011–2012.

### Statistical Analysis

Statistical evaluation of the percentages of incidence was performed by means of a chi-square test. Statistical significance was set for *p*-values < 0.05.

## 3. Results

The total numbers of births in the Umbria region were 5378 and 5359, respectively, in 2020 and 2021. Table 1 summarizes the UNHS activities conducted in the Umbria region in the two years taken into consideration (2020–2021) and the same activities conducted in 2011 and 2012, according to the study conducted by Molini et al. [10]. Based on the report, in 2020 99.52% of WB and 97.56% of BRF correctly performed UNHS tests within 1 month of birth. In 2021, this percentage reached 99.5% for WB and 95.93 for BRF. Reports show that 98.91% in 2020 and 98.64% of WB tested passed the TEOAE testing; 93.29% in 2020 and 87.76% in 2021 of BRF tested passed the TEOAE and A-ABR testing. WB who failed the UNHS tests (“refer”) in 2020 and 2021 were 1.09% and 1.36%, respectively, while among the BRF refer this number rose from 6.71% in 2020 to 14.69% in 2021. The percentage of babies lost to follow-up, who did not show up for the identification or diagnostic level, increased from 31.16% in 2020 to 36,13% in 2021. The number of WB with hearing loss diagnosed was 5 in 2020 and 7 in 2021, while for BRF, hearing loss was diagnosed in 10 cases in 2020 and 16 in 2021. Table 2 summarizes the mean age of diagnosis of hearing loss for WB and BRF, compared to that found in the three-year period 2010–2012. The grey-background boxes of the tables also highlight the values that do not meet the benchmarks. 

## 4. Discussion

The analysis of the results of the regional application of UNHS provides a detailed cross-section of the local health situation, especially interesting from the perspective of its evaluation during the COVID-19 pandemic. Prior to the institution of universal newborn hearing screening, the mean age of hearing impairment diagnosis was 26 months, with subsequent hearing aid treatment at 32.2 months [11].

Our study has shown how, with the implementation of the UNHS, the coverage rate of WB screening, despite the difficulties related to the pandemic, has significantly (*p* < 0.0001) increased from 93.40%, on average, in the two-year period 2011–2012, to 99.69%, on average, in the two-year period 2020–2021, to currently meet the JCIH benchmark (95%) [9], and has remained stabilized well above the desired values throughout the critical period of the pandemic. Instead, with regard to BRF, this data is significantly lower in the two-year period 2011–2012 (*p* < 0.00001) because in this period, in the collection of data, babies that were considered to have audiological risk factors were only those coming from the neonatal intensive care unit (NICU). In fact, with a greater number of births, the number of children with the risk factor is lower; therefore, although the percentage of of those tested is lower for 2020–2021, the ability to identify risk factors has drastically increased. The total number of BRF has risen over the years (from 3.61%, on average, in 2011–2012 to 7.95%, on average, in 2020–2021). There have been changes in the JCIH recommendations, but perhaps this aspect also reflects a greater sensitivity and attention to hearing and the conditions that require the monitoring of a child’s growth.

Since screening became a part of clinical practice at birth centers, qualified personnel have been trained adequately every day, as opposed to the early years of its introduction when screening was viewed as a new practice. As a result of the Essential Level of Assistance (ELA) of 2017 that made screening mandatory and introduced data collection on a regional platform, hearing screening has become a regular part of infant care. One of the reasons for greater efficiency and coverage could also be related to the drastic decline in the birth rate in Umbria, which may have reduced the workload at the nursery. Indeed, a reduction in live births from about 8000 in 2011–2012 to about 5000 in 2020–2021 has been observed.

The percentage of WB referrals, which significantly decreased (*p* < 0.0001) between the years 2011–2012 and the COVID-19 period, is another indicator of the screening program’s greater efficiency in the Umbria region. The authors link the results to the greater experience that nurses have acquired over the years in addition to the present use of TEOAE instruments that are entirely automatic, rapid and easy to use. 

However, a critical issue has emerged: the most penalized phase in the screening process is access to the diagnostic phase. Despite a global improvement in the efficiency of screening, there are still too many children who do not reach the second-level assessment, where the hearing deficit is eventually identified. The percentage of those lost to follow-up has always remained greater than 30%, reaching an unacceptable level since 2011, well above the benchmark. 

This is a well-known issue, shared by colleagues involved in hearing screening nationally and internationally [12,13,14,15]. From the literature, it emerges that parental involvement is crucial in appointment cancellations and delays, as well as resistance to assessments and therapies [16]. According to Feresin et al. [17], “document-miss” is one of the most frequent types of “missing” cases, due to inefficiency in data documentation for newborns in the Friuli–Venezia Giulia region.

Analyzing the lost-to-follow-up population in our study, it is possible to identify that the majority of those who miss screening are in fact newborns with risk factors who, before discharge, are only able to undergo the TEOAE test. It can be deduced from the available data that completing the screening process is most difficult in cases of those who passed the test at birth. It should be recalled that it is not possible to perform A-ABR at all birth points: this, therefore, implies the need for families of children with audiological risk factors to reach a level II center to complete the screening. On the other hand, the number of those who are not tested at birth or who, having been categorized as REFER, do not continue the screening process is very low. 

The high rate of those lost to follow-up is probably also attributable to the pandemic’s effects on the patient–hospital interaction. The COVID-19 infection has undoubtedly inhibited this procedure. Considering the enhancement of our region’s whole hearing screening procedure, it is reasonable to think that, in other circumstances, the lost-to-follow-up rate observed would probably have aligned with, or in any case approached, the 10% benchmark identified and recommended by the JCIH. Compared to the years 2010–2012, the age of diagnosis has significantly reduced for WB and BRF. In the WB population, a diagnosis was reached in 2.16 months on average in 2020, and in 2021 the average was 5.23 months. In detail, in 2021, on average, 6.8 months were needed for the diagnosis of unilateral hearing loss, while for the diagnosis of bilateral hearing loss 4.97 months were required. In the BRF population, the average age at diagnosis was 3.76 months in 2020 and 4.04 months in 2021. However, by distinguishing the diagnoses of unilateral or bilateral hearing loss, it can be seen that in the first case the average age at diagnosis is 4.54 months, while in the bilateral forms the average age is 3.33 months. The age at diagnosis is probably also affected by the COVID-19 pandemic. It is also emphasized that the average age at diagnosis has varied more significantly in the group of those who do not have risk factors, for which, precisely because of the pandemic, the control period has been extended. A recent commentary by John Hopkins University pointed out that a high number of diagnoses of late hearing loss is to be expected in the coming years. This is due to intra-hospital reorganizations and staff decreases that have necessarily overshadowed some outpatient services, and also due to the increase in home births and the population’s fear of accessing hospital services [18]. 

Even if not always within the benchmarks, the timing approaches the threshold for which the JCIH promotes a “quality leap”, and that is time to think of 1-2-3 times instead of 1-3-6. 

## 5. Conclusions

The results of our study show that there has been a clear improvement in the implementation of the screening program in the Umbria region. It was possible to satisfy most of the quality indicators and benchmarks provided by the JCIH, although we are still far from satisfying those relative to the lost-to-follow-up population. As far as the COVID-19 pandemic has certainly had a significant impact on this data, we believe it is necessary to carefully monitor those who do not access the diagnostic level, in order to identify all the causes of loss to follow-up, and therefore also to be able to meet this benchmark in the future. To emphasize the importance of a proper screening program, while the effects of the COVID-19 pandemic fade, it will be helpful to strengthen the computerized data collection system and create an information network between audiologists, pediatricians and families.

## Figures and Tables

**Table 1:** UNHS activities conducted in the Umbria region in 2020–2021.

	LIVE BIRTHS	TOT BRF (%)	TOT WB Tested (%)	TOT BRF Tested (%)	TOT WB PASS (%)	TOT BRF PASS (%)	REFER WB	REFER BRF	TOT REFER WB+BRF (%)	TOT Lost to Follow-Up (%)	HL WB	HL in BRF
2020	5378	411 (7.64)	4943 (99.52)	401 (97.56)	4889 (98.91)	320 (93.29)	54 (1.09)	23 (6.71)	77 (1.45)	24 (31.16)	5 (0.101)	10 (2.91)
2021	5359	443 (8.27)	4909 (99.86)	425 (95.93)	4842 (98.64)	373 (87.76)	67 (1.36)	52 (14.69)	119 (2.26)	43 (36.13)	7 (0.142)	16 (3.76)
2011	8213	300 (3.65)	7346 (92.83)	300 (100)	6312 (85.92)	283 (94)	1034 (14.07)	17 (5.66)	1051 (13.74)	331 (31.49)	18 (0.245)	9 (3.0)
2012	8229	294 (3.57)	7458 (93.98)	294 (100)	6690 (89.7)	256 (87.07)	768 (10.29)	38 (12.92)	806 (10.39)	275 (34.11)	18 (0.241)	20 (8.03)

TOT = Total. HL = hearing loss. WB = well born babies. BRF = babies with identified audiological risk factors. LIVE BIRTHS = total number of live births in the year; TOT BRF = total number of babies that presented a neonatal risk (based on JCIH, 2019); TOT WB Tested = total number of babies who performed the TEOAE screening in the well baby clinic; TOT BRF Tested = total number of at-risk (JCIH) babies who performed the TEOAE/A-ABR screening; TOT WB PASS = total number of well born babies who passed the TEOAE screening; TOT BRF PASS = total number of at-risk (JCIH) babies who performed the TEOAE/A-ABR screening; REFER WB = total number of well born babies who failed on one or both sides the TEOAE screening; REFER BRF = total number of at-risk (JCIH) babies who failed on one or both sides the TEOAE/A-ABR screening; TOT REFER WB+BRF = sum of REFER WB and REFER BRF; TOT lost to follow-up = total number of children lost to follow-up, i.e., who did not show up for the identification or diagnostic level; HL WB = total number of children that were affected by permanent hearing loss among the well born babies (the percentage refers to the total n of WB tested); HL in BRF = total number of at-risk children that were affected by permanent hearing loss. The percentage refers to the total number of BRF tested. The grey-background boxes highlight the values that do not meet the benchmarks.

**Table 2:** Mean age of diagnosis of hearing loss for WB and BRF, a comparision between 2020-2021 and 2010–2012.

	Tot HL in WB	Mean Age at Diagnosis	HL in BRF	Mean Age at Diagnosis
2020	5	2.16	10	3.76
2021	7	5.23	16	4.04
2010–2012 (3 years)	40	5.31 (+/−3.95)	34	11.29 (+/−7.73)

HL= hearing loss. WB = well born babies. BRF= babies with identified audiological risk factors. The grey-background boxes highlight the values that do not meet the benchmarks.

## Data Availability

The data presented in this study are available on request from the corresponding author.
